# Evaluation of plant growth promoting activity and heavy metal tolerance of psychrotrophic bacteria associated with maca (*Lepidium meyenii* Walp.) rhizosphere

**DOI:** 10.3934/microbiol.2017.2.279

**Published:** 2017-05-02

**Authors:** Paola Ortiz-Ojeda, Katty Ogata-Gutiérrez, Doris Zúñiga-Dávila

**Affiliations:** Laboratorio de Ecología Microbiana y Biotecnología, Departamento de Biología, Universidad Nacional Agraria La Molina, Lima, Peru

**Keywords:** andean, psychrotrophs, heavy metals, PGPR, germination

## Abstract

The high Andean plateau of Peru is known to suffer harsh environmental conditions. Acidic soils containing high amount of heavy metals due to mining activities and withstanding very low temperatures affect agricultural activities by diminishing crop quality and yield. In this context, plant growth promoting rhizobacteria (PGPR) adapted to low temperatures and tolerant to heavy metals can be considered as an environment-friendly biological alternative for andean crop management. The aim of this work was to select and characterize psychrotrophic PGPR isolated from the rhizosphere of maca (*Lepidium meyenii* Walp.) a traditional andean food crop. A total of 44 psychrotrophic strains isolated from 3 areas located in the Bombon plateu of Junin-Peru were tested for their PGPR characteristics like indole acetic acid (IAA) production, phosphate solubilization and for their ability to improve seed germination. In addition, their capacity to grow in the presence of heavy metals like cadmium (Cd), lead (Pb), cobalt (Co) and mercury (Hg) was tested. Of the total number of strains tested, 12 were positive for IAA production at 22 °C, 8 at 12 °C and 16 at 6 °C. Phosphate solubilization activities were higher at 12 °C and 6 °C than at 22 °C. Red clover plant assays showed that 16 strains were capable to improve seed germination at 22 °C and 4 at 12 °C. Moreover, 11 strains showed tolerance to Cd and Pb at varying concentrations. This study highlight the importance of obtaining PGPRs to be used in high andean plateu crops that are exposed to low temperatures and presence of heavy metals on soil.

## 1. Introduction

The Bombon plateu is located on the high Andes of Junin, Peru. It is known for its dry climate, extremely low temperatures and acidic soils (pH < 5) with relatively high concentration of toxic heavy metals. Among these, low temperatures and soil contamination are common problems which affect farmers of this region, together, these two factors diminish the yield of economically important crops such as maca (*Lepidium meyenii* Walp.) a Brassicaceae whose roots are used as food. Quality of the crop is also affected reducing its demand in international markets.

The soil is a habitat for a wide variety of microorganisms. The rhizosphere, an area surrounding plant roots, is a special ecological niche that supports a vast group of metabolically versatile microorganisms [1]. Among these, plant growth promoting rhizobacteria (PGPR) are considered of great importance due to their ability to solubilize inorganic phosphates, transport iron, produce phytohormones like indole acetic acid (IAA) [2] and fix atmospheric nitrogen [3]. These attributes allow those microbes to induce plant growth and development. In this study, PGPRs can be considered as a biological alternative to improve the yield and growth of crops growing in the high Andes under harsh environmental conditions. In this context, the aims of this research were to select and characterize psychrotrophic bacteria isolated from maca rhizosphere by testing their plant growth promoting ability and tolerance to heavy metals. There are only few studies that explore the application of biofertilizers based on psychotrophic bacteria with PGPR characteristics, adapted to the harsh climatic conditions of the high Andean region and with potential to bioremediate soils contaminated with heavy metals.

## 2. Materials and Methods

### 2.1. Sampling and isolation of bacteria

Soil samples were collected from fields in the Comunidad Campesina San Pedro de Cajas, Province of Tarma-Junin (located at 4100 meters above sea level). Maximum and minimum soil temperatures were 22 °C and 6 °C, respectively, with a relative humidity of 55.2%. Three locations were sampled: Galpón Condorín (Longitude W 75°53′49.54″ Latitude S 11°15′12.18″), Acomachay (Longitude W 75°53′38.64″ Latitude S 11°14′15.24″) and Condorín (Longitude W 81°23′42″ Latitude S 82°7′32.01″) which were either at the stage of first harvest, second harvest and 10 years of land fallow (without planting any crop), respectively. Rhizosphere and soil samples were collected from different plants. Each plant was taken completely along with their associated soil. Moreover, 500 g of soil was also collected from the area lacking any plant material. Samples were processed later in accordance with the methodologies established by APHA (1998) [4] for isolation of viable aerobic bacteria. Temperature and time of incubation was modified to 6 °C for 7 days to obtain isolates with the ability to grow at low temperatures. Rhizosphere and soil samples were also characterized for their physico-chemical properties like pH, conductivity, texture, organic matter, nutrients, among others.

### 2.2. Bacterial growth at different temperatures

Isolates were grown in Nutrient Agar (NA) medium and incubated at 6 °C, 12 °C, 22 °C and 28 °C [5] in order to determine their nature as psychrotrophic or psychrophilic bacteria. Growth was evaluated after 7 days of incubation and results were expressed on a scale of 1 to 4 (where 4 represent the maximum colony growth).

### 2.3. Characterization of IAA production

IAA production was estimated using the colorimetric technique based on the Salkowski reagent [6]. Strains were grown in 5 ml of Yeast Extract Mannitol (YEM) broth suplemented with 5 mM L-tryptophan and were incubated at 6 °C, 12 °C and 22 °C for 28, 19 and 11 days, respectively. Salkowski reagent (1:4) [7] was added to each culture media and the mix was kept in the dark for 30 min. Positive samples turned from shades of pink to intense rose. Optical density (OD) was registrated at 530 nm to measure IAA production. The amount of IAA in µg/ml was calculated by plotting a standard curve [8].

### 2.4. Phosphate solubilization capacity of bacteria

Isolates were grown in YEM broth and incubated at 6 °C, 12 °C and, 22 °C for 28, 19 and 11 days, respectively. Five µl of each culture at a concentration of 10^8^ CFU/ml [9] was inoculated in National Botanical Research Institute's Phosphate (NBRIP) basal medium plates supplemented with di-calcium (CaHPO_4_) or tri-calcium (Ca_3_(PO4)_2_) phosphate [10]. Plates were incubated at 6 °C and 12 °C for 36 days and at 22 °C for 8 days. Strains with a translucent halo around the colony were considered positive for phosphate solubilization.

### 2.5. Seed germination assay

Strains for this assay were selected according to positive results obtained on at least one of the *in vitro* PGPR traits. Red clover (*Trifolium pratense*) seeds were used as a model plant to evaluate the efficacy of the isolates on germination promotion. Disinfection and inoculation of seeds (10^6^ UFC/ml) was carried out following the methodology described by Ogata and Zúñiga [12]. Seeds were incubated at 12 °C or 22 °C after inoculation. Inoculation with water was used as control. Each experimental unit consisted of 25 inoculated seeds in a water agar (0.75%) plate. Four replicates were used for each treatment. The percentage of germination was evaluated with the following formula:

$$\text{\%G}=\frac{\text{Number of germinated seeds}}{\text{Total number of seeds}}\times \text{100}$$

### 2.6. Heavy metal resistance

All strains tested in the germination assay were also evaluated for their ability to grow in the presence of four heavy metals: Pb (lead trihydrate), Cd (cadmium chloride), Co (cobalt chloride) and Hg (mercury chloride II). The assay was conducted following the method described by Lalitha [13] using 0.5 mM Cd, Pb or Co and 0.01 mM Hg [14]. In addition, a mixture of all metals at 0.5 mM was also used. Strains resistant to the different metals tested were further evaluated for their minimum inhibitory concentration (MIC) using Pb and Cd separately. The assay was conducted using 8 concentrations (0.005, 0.01, 0.05, 0.1, 0.5, 1, 2.5 and 5 mM) of each heavy metal on NA medium adjusted to pH 5 [14] and incubated at 6 °C for 30 days.

### 2.7. Molecular identification and phylogenetic analysis

Molecular identification was carried out using 16S rDNA gene polymerase chain reaction (PCR) amplification and sequencing by using primer pair fD1 (5′-CCGAATTCGTCGACAACAGAGTTTGATCCTGGCTCAG-3′) and rD1 (5′-CCCGGGATCCAAGCTTAAGGAGGTGATCCAGCC-3′) [15]. The ClustalX2 software [16] was used for alignment of sequences generated in this study along with sequences obtained from databases. Phylogenetic relationships were established with the neighbor-joining (NJ) method and distances calculated according to the Kimura-2 parameter model using the MEGA6 software [17].

## 3. Results and Discussion

### 3.1. Isolation of PGPR

Physicochemical characterization of the soils samples revealed that they were non-saline, moderately thick with sandy loam consistency and acidic (pH values ranging from 4 to 5). Concentrations of the metals such as B, Fe, Mn, Cu, Zn and Cr were in the range of 13.86–18.87 ppm. The concentration of lead (Pb) was in the range of 33.92–56.14 ppm while that of cadmium (Cd) was between 1.43–1.98 ppm. Based on the Environmental Quality Standards of agricultural soils, the samples slightly exceded the maximum permissible values of Cd but not of Pb. According to the Peruvian normative, concentrations under 70 ppm and 1.4 ppm of Pb and Cd, respectively, are allowed for agricultural soil.

**Figure 1.**
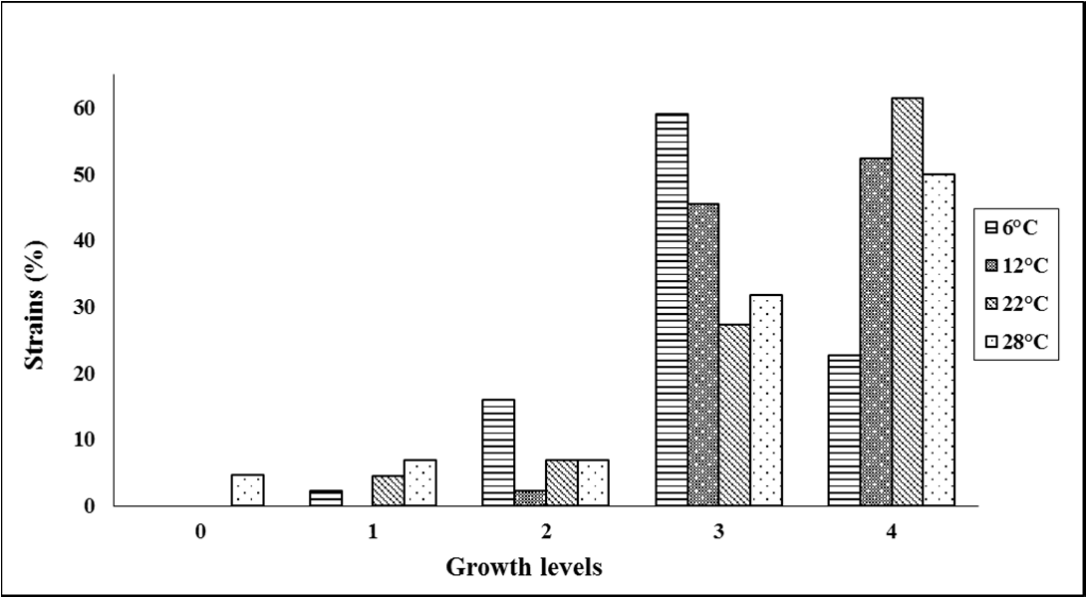
Percentage of strains showing different growth levels (1–4) at different temperatures (6 °C, 12 °C, 22 °C, 28 °C).

A total of 44 bacterial strains were isolated: 8 from Galpón Condorín (1st year of harvest), 30 from Acomachay (2nd year of harvest) and 6 from Condorin (10 years of rest). Twenty three were isolated from the rhizosphere and 21 from non-rhizospheric soil. Growth at different temperatures, showed that more than 59% of the strains have a growth level of 4 when they were incubated at 12 °C, 22 °C and 28 °C (Figure 1). All isolates grew at 12 °C and 6 °C. These results may be related to their origin in the high andean zone, where soils reach very low temperatures (close to 0 °C) during the freezing season. Their versatility to grow at different temperatures indicated that the strains belong to the psychrotrophic group (Przemieniecki et al., 2014). It is worth noting that strains obtained in this study took 7 days to grow under low temperatures while Calvo and Zúñiga (2010) reported that strains belonging to the genus *Bacillus* took only 4 days to grow under the same conditions.

### 3.2. IAA production assays

Strains were tested for their ability to produce IAA at three temperatures i.e. 22 °C, 12 °C and 6 °C (Table 1). Among the tested strains, LMTK39 was able to produce the maximum amount of the phytohormone at 22 °C and 12 °C, 60.6 µg/ml and 31.1 µg/ml, respectively. Whereas strain LMTK11 showed the highest ability to produce IAA at 6 °C (14.1 µg/ml). It was observed that the number of strains having the ability to produce IAA at 6 °C were higher than that of those at 22 and 12 °C. The above finding was not unexpected due to the fact that the amount of IAA biosynthesis varies as per the nature of the strain and environmental factors like altitude, temperature, drought conditions etc. In addition, a number of other factors related to stress conditions affecting these microbes are also known to influence with IAA biosynthesis [2],[20]. Subramanian et al. (2016) [21] reported that bacterial strains isolated from soils collected in regions with an average temperature of about 2 °C, have the ability to produce IAA at 5 °C. IAA production by these strains was found to be in the range of 0.3 to 17 µg/ml, values which are similar to those found in this study at 6 °C. A similar test was also performed by Zúñiga et al. (2011) [22] with strains isolated from maca rhizosphere at 28 °C showing less IAA production ability (0.3–11.9 µg/ml) at 5 °C than those reported in this study.

**Table 1.** PGPR traits of the strains used in this study. IAA (µg/ml) production and halo of phosphate solubilization (mm).

STRAINS	IAA (µg/ml)			CaHPO_4_ (mm)			Ca_3_(PO_4_)_2_ (mm)		
	22 °C	12 °C	6 °C	22 °C	12 °C	6 °C	22 °C	12 °C	6 °C
LMTK1	–	–	3.6	–	–	–	–	–	–
LMTK4	–	–	3.3	–	–	–	–	–	–
LMTK5	–	–	4.0	–	–	–	–	–	–
LMTK6	2.9	–	3.2	1.25	5.25	–	–	2	–
LMTK7	7.0	3.0	3.2	–	–	–	–	–	–
LMTK10	1.8	4.8	3.2	–	–	–	–	–	–
LMTK11	5.4	15.1	14.1	11.5	23.5	18	2	2.5	2.75
LMTK15	–	–	4.5	–	–	–	–	–	–
LMTK25	1.8	0.9	6.9	–	–	–	–	–	–
LMTK28	3.7	–	0.9	8	20.5	11.25	–	2.5	–
LMTK32	2.0	–	1.1	8	19.25	11	–	2.5	2.5
LMTK34	12.3	–	4.7	7	20.5	10	–	2	4.5
LMTK36	–	–	–	9	17	12.5	–	2	1
LMTK37	12.8	6.0	12.1	–	–	–	–	–	–
LMTK39	60.6	31.1	12.5	–	–	–	–	–	–
LMTK42	0.4	1.5	12.6	8	16.25	12.25	–	1.25	1
LMTK43	3.0	9.8	9.5	3	6.25	3.25	–	1.25	1.5

### 3.3. Phosphate solubilization ability

Eight strains were able to solubilize di-calcium and tri-calcium phosphate. Strain LMTK11 was the only one which had the ability to solubilize tri-calcium phosphate at 22 °C. This strain showed a solubilization halo of 2 mm, while in a di-calcium phosphate media it was capable to produce a halo size of 11.5 mm. Solubilization halo of the strains from this study varied from 5.25 mm to 23.5 mm at 12 °C. Strains LMTK11, LMTK28 and LMTK34 showed the maximum ability to solubilize di-calcium phosphate at this temperature. The ability of the strains to solubilize di-calcium at 6 °C was lower than at 12 °C and higher than at 22 °C. At this temperature (6 °C), strains LMTK11, LMTK28 and LMTK34 displayed solubilization halos of 18, 11.3 and 10 mm respectively (Table 1). In a similar study, strains isolated from the Himalayas and living at harsh conditions of low temperature and high altitude, showed a range of solubilization efficiency between 25.8–375.4 and 31.7–456.5 at incubation temperatures of 4 and 10 °C, respectively [23]. From all the strains tested, *Pseudomonas* sp. showed high ability to solubilize di-calcium and tri-calcium phosphates. Mishra et al. (2015) [24] also reported *Pseudomonas* sp. as the bacterial genus with the highest solubilization activity. In contrast, Zúñiga et al. (2011) [22] found that free-living diazotrophs showed the best solubilization halos in di-calcium and tri-calcium media at temperatures of 5, 14 and 28 °C.

### 3.4. Promotion of seed germination

**Figure 2.**
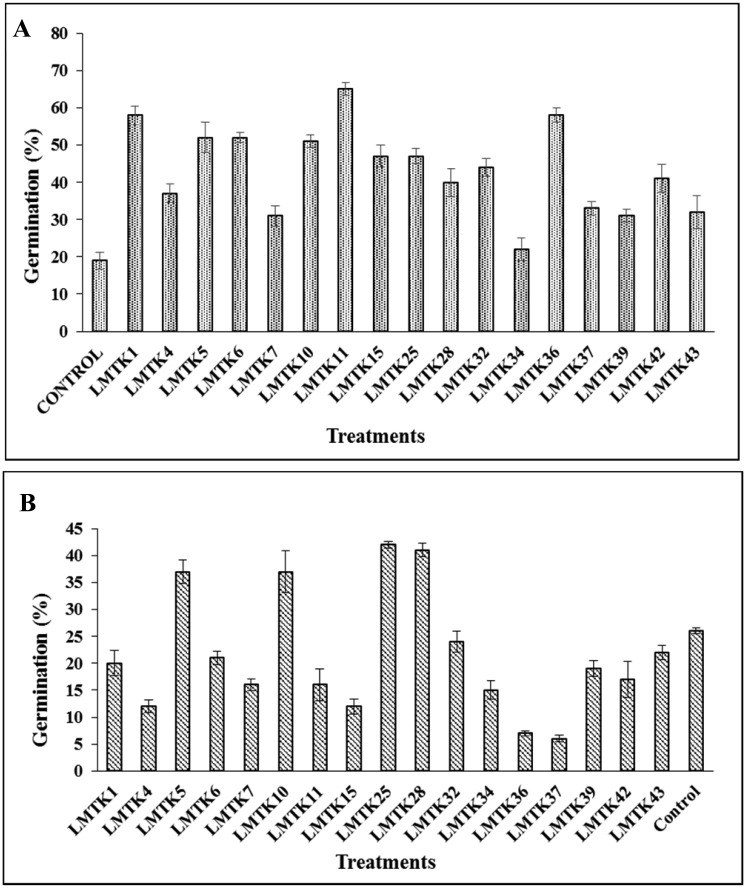
Effect of inoculation with different bacterial strains on red clover seed germination. A. 22 °C; B. 12 °C.

Sixteen from the seventeen strains tested in the red clover germination assay at 22 °C significantly increased the percentage of germination compared to the non-inoculated control (Figure 2A). LMTK11 strain showed the best performance in the germination assay at this temperature. These results are consistent with those reported by Kloepper et al. (1991) [25], which emphasize the role of PGPR in improving seed germination rates through the production of plant growth regulating substances such as auxins, cytokinins or gibberellins [26]. The majority of the strains able to increase the germination percentage were also positive for both IAA production and phosphate solubilization. Although, some of the strains did not show high values in all PGPR traits, they were able to improve seed germination in comparison to the non-inoculated control. At 12 °C, strains LMTK25 and LMTK28 showed better ability to increase the germination rate (42 and 41%, respectively) than the control (26%) (Figure 2b). A study conducted by Acosta et al. (2012) [27] also higlighted the importance of incubation temperature for the induction of seed germination.

### 3.5. Bacterial growth at different heavy metal concentrations

Eleven out of the seventeen strains tested were able to grow in the presence of different heavy metals (0.5 mM Pb, Cd and Co; 0.01 mM Hg). It was shown that the strains were able to better tolerate the heavy metals when they were cultivated at pH 5 rather than 4 (Table 2). Other studies show a variable effect of heavy metals such as lead and cadmium in different organisms, demonstrating that the toxicity depends not only on pH but also on the concentration and organism evaluated. [28],[29],[30]. Strain LMTK11 was the only one able to grow at both pH 4 and 5 in a culture media supplemented with Hg; while in media supplemented with Co at pH 5, the growth of strains LMTK11, LMTK32 and LMTK36 was the only observed. Interestingly, strain LMTK11 was able to grow in NA plates supplemented with a mixture of heavy metals (Pb, Cd, Co and Hg). Mechanisms reported for heavy metal resistance in bacteria are: (1) exclusion of metal by a permeable barrier, (2) exclusion by active transport of the metal from the inside of the cell outwards, (3) intracellular physical sequestration of the metal using a polymer to prevent metal cellular damage, (4) extracellular sequestration, (5) enzymatic detoxification of the metal to a less toxic form, and (6) cell reduction of the sensitivity to metals [31].

### 3.6. Minimum inhibitory concentration assay

Minimum inhibitory concentration (MIC) assays was performed using Pb and Cd. Based on the results obtained, MIC values of the 11 selected strains were calculated to be 0.5 mM and 0.05 mM for Pb and Cd, respectively (Table 3). Kumar et al. (2015) [32] reported two *Bacillus* strains with MIC values of 250 and 1000 ppm for Pb. On this kind of assay, reduction in colony size is common. This reduction is attributed to the toxic effects that both metals exert on bacterial cell affecting its metabolism, causing a blockage of enzymes and polynucleotides activities [33],[34]. These heavy metals are also known to interfere with the transport of essential nutrients and ions. They also affect organisms by either substitution or displacement of essential ions in the cell or through denaturation and inactivation of key enzymes. In addition, heavy metals are able to affect bacterial membrane integrity [31]. Although high concentrations of heavy metals inhibit bacterial growth, there are some species which are resistant and could grow up to 5000 ppm of Pb [35]. In course of the assays, it was noticed that some bacterial colony sizes increased as the concentration of the metals were increased. Strain LMTK43 showed an increase in colony size in comparison to the control devoid of heavy metals. Some studies have described the ability of certain strains to use these metals as growth enhancers. For instance, in a study conducted by Azario et al. 2010 [36], significant increase in growth of *E. coli* ATCC 35218 was observed when it was cultivated in a media supplemented with Cr, Cd and Pb separately or together, as a cocktail.

**Table 2.** Growth ability of eleven strains in the presence of different heavy metals at pH 4 and 5.

STRAINS	pH 4					pH 5				
	Cd	Pb	Co	Hg	MIX	Cd	Pb	Co	Hg	MIX
**LMTK5**	x					x	x			
**LMTK11**	x			x	x	x	x	x	x	x
**LMTK28**	x					x	x			
**LMTK32**	x					x	x	x		
**LMTK33**	x					x	x			
**LMTK34**	x					x	x			
**LMTK36**	x					x	x	x		
**LMTK37**							x			
**LMTK39**							x			
**LMTK42**	x					x	x			
**LMTK43**	x	x				x	x			

x, Growth ability; MIX, mixture of different heavy metals.

**Table 3.** Strain tolerance percentages to different concentrations (mM) of lead and cadmium.

Heavy metals	lead and cadmium concentrations (mM)							
	0.005	0.01	0.05	0.1	0.5	1	2.5	5
**Pb**	100	100	100	100	72.7	0	0	0
**Cd**	100	100	72.7	63.6	63.6	54.5	0	0

### 3.7. Phylogenetic analysis of the strains

The NJ phylogenetic tree (Figure 4) showed that the studied strains were clustered in 8 groups distributed in the *Pseudomonas*, *Bacillus, Paenibacillus, Sporosarcina, Cupriavidus* and *Paenarthrobacter* genera. The majority of strains belonged to *Bacillus* and *Pseudomonas*. *Bacillus* and *Pseudomonas* genera are usually reported as PGPR and are found to be dominant in bacterial-plant studies [37]. Among the Firmicutes, five strains were clustered within the *Bacillus simplex* group and two strains were grouped with *Paenibacillus sinodophylli.* One strain of Actinobacteria grouped with *Paenathrobacter nitroguajacolicus*. Proteobacteria were represented by one strain related to *Cupriavidus gilardii*, one strain to *Pseudomonas taiwanensis* while five strains were grouped with *Pseudomonas constaninii*. The comparison of the 16S rDNA gene sequence of all the isolates against type strains of bacterial species recorded in the EzCloud database is shown in Table 4.

**Table 4.** Comparison of 16S rDNA sequences obtained from strains isolated in this study and related type strains.

Strains	Molecular identification using 16S rDNA gen	Accession number	Similarity (%)
LMTK1	*Bacillus simplex* NBRC 15720^T^	BCVO01000086	99.8
LMTK4	*Bacillus simplex* NBRC 15720^T^	BCVO01000086	99.8
LMTK5	*Bacillus simplex* NBRC 15720^T^	BCVO01000086	99.8
LMTK6	*Paenarthrobacter nitroguajacolicus* G2-1^T^	AJ512504	99.8
LMTK7	*Bacillus butanolivorans* DSM 18926^T^	LGYA01000001	99.8
LMTK10	*Bacillus butanolivorans* DSM 18926^T^	LGYA01000001	99.8
LMTK11	*Pseudomonas fluorescens* DSM 50090^T^	LHVP01000014	99.5
LMTK15	*Bacillus simplex* NBRC 15720^T^	BCVO01000086	99.8
LMTK25	*Bacillus simplex* NBRC 15720^T^	BCVO01000086	99.8
LMTK28	*Pseudomonas trivialis* DSM 14937^T^	JYLK01000002	99.2
LMTK32	*Pseudomonas simiae* OLi^T^	AJ936933	99
LMTK34	*Pseudomonas simiae* OLi^T^	AJ936933	99
LMTK36	*Pseudomonas simiae* OLi^T^	AJ936933	99.5
LMTK37	*Paenibacillus sinopodophylli* TEGR-3^T^	KX009022	98.2
LMTK39	*Paenibacillus sinopodophylli* TEGR-3^T^	KX009022	96
LMTK42	*Cupriavidus gilardii* LMG 5886^T^	AF076645	99.5
LMTK43	*Pseudomonas baetica* a390^T^	FM201274	99.5

**Figure 3.**
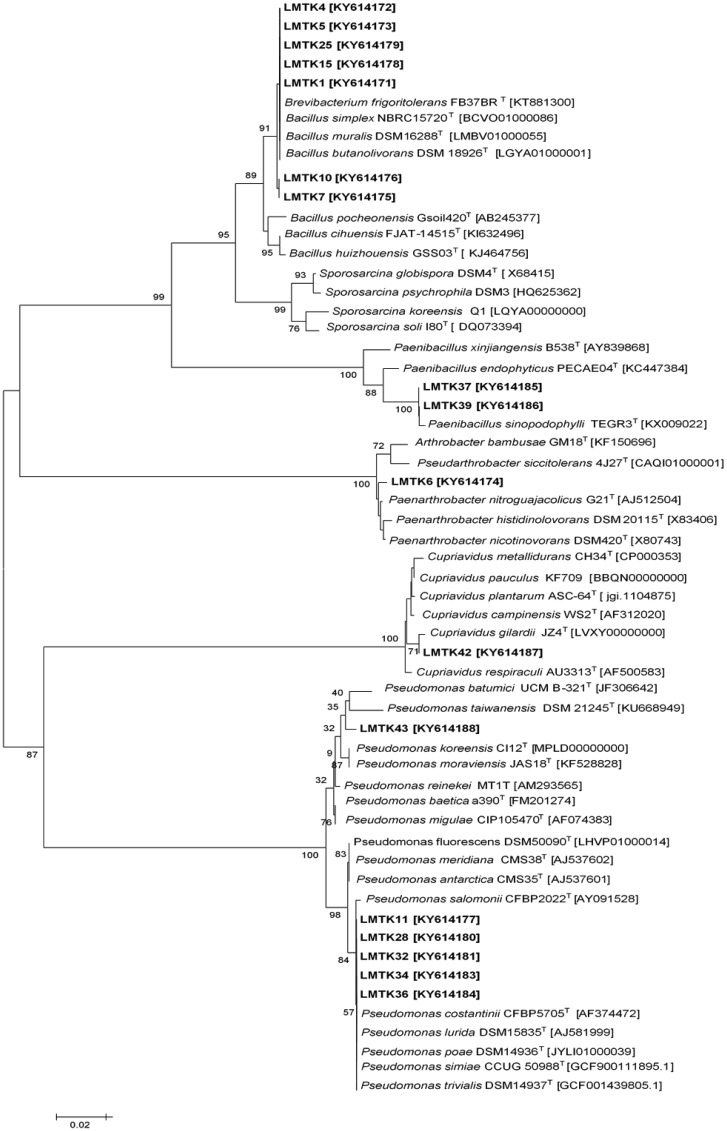
Phylogenetic tree based on 16S rDNA sequences. Strains obtained in this study are indicated in bold. Only bootstrap values greater than 60% are shown (1,000 pseudoreplicates). Type strains of related species were obtained from Ez-biocloud.

## 4. Conclusions

The results obtained in this study demonstrate the PGPR potential of these strains; as well as their ability to grow in the presence of heavy metals such as lead and cadmium under *in vitro* conditions. This study provides first experimental evidence highlighting the potential of these strains as biofertilizers and bioremediators for the high Andean soils, due to their ability promote plant growth, ability to grow at low temperatures and to tolerate heavy metal toxicity.
